# The Efficacy and Safety of Thiopurines Do Not Differ Between Geriatric and Non-Geriatric Patients with Inflammatory Bowel Disease

**DOI:** 10.7150/ijms.116202

**Published:** 2026-01-01

**Authors:** Derya Arı, Kerem Kenarlı, Ferhat Bacaksız, Sinan Arı, Volkan Gökbulut, Ömer Öztürk, Yasemin Özderin Özin, İsmail Hakkı Kalkan

**Affiliations:** 1 Ankara Bilkent City Hospital Department of Gastroenterology, Turkey.; 2 Diyarbakır Gazi Yaşargil Training and Research Hospital, Department of Gastroenterology, Turkey.; 3 Ankara Training and Research Hospital, Department of Geriatric Medicine, Turkey.; 4 TOBB University of Economics and Technology, School of Medicine, Department of Gastroenterology, Turkey.

**Keywords:** elderly, Crohn's disease, ulcerative colitis, inflammatory bowel disease, immunomodulatory therapy

## Abstract

**Background and Aim:** The patterns of medication use among elderly patients with inflammatory bowel disease vary owing to several factors. This study aimed to present the outcomes of immunomodulator therapy in both geriatric and non-geriatric patient groups, highlighting the differences in treatment effectiveness, reasons for discontinuation, and incidence of side effects between the two groups.

**Materials and Methods:** A retrospective database was established by reviewing the records of patients with inflammatory bowel disease aged 18 years and older who received immunomodulatory therapy at our clinic over a seven-year period. Patients aged 65 years or older were included in the geriatric cohort. In addition to demographic and clinical variables, disease activity in ulcerative colitis patients was assessed using the Mayo Disease Activity Index and the Rachmilewitz Endoscopic Activity Index, whereas the Harvey-Bradshaw Index was used for Crohn's disease patients.

**Results:** This study included 120 patients, comprising 52 with ulcerative colitis and 68 with Crohn's disease. The median age was 66.5 years (range: 23-87), with 64 patients (53.3%) classified as geriatric (≥65 years). Immunomodulator therapy was discontinued owing to side effects in 37 patients (30.8%) and remission in 13 patients (10.8%). In addition, 29 patients (24.2%) did not respond to the treatment. During follow-up, 3 patients (2.5%) developed malignancy and 1 patient (0.8%) died. No significant differences were observed between the geriatric and non-geriatric groups regarding the type of inflammatory bowel disease, duration of therapy, concomitant treatments, reasons for discontinuation, side effects, occurrence of malignancy, malignancy type, or mortality.

**Conclusion:** With careful patient selection and close monitoring, immunomodulator therapy in elderly patients with inflammatory bowel disease can achieve similar effectiveness and risk of side effects as those observed in younger patient groups.

## Introduction

Inflammatory bowel disease (IBD), a chronic inflammatory disorder of the gastrointestinal tract, is characterized by two primary subtypes: Crohn's disease (CD) and ulcerative colitis (UC). Although traditionally considered a condition of younger individuals, 10-30% of IBD patients are over 60 years old, either aging with the disease or developing it later in life 1. It is unsurprising that the incidence and prevalence of IBD among geriatric individuals are increasing, given the global increase in IBD incidence and the aging population.

Epidemiological research indicates that late-onset IBD predominantly affects the colon, tends to follow a milder clinical course, and presents with fewer extraintestinal manifestations. However, elderly patients face heightened mortality risk due to comorbid conditions, polypharmacy, and reduced resilience to severe disease progression. In addition to the distinct pathophysiology of IBD in elderly adults, these patients encounter numerous challenges related to its management. Elderly adults with IBD are frequently excluded from clinical trials due to factors such as advanced age, multiple comorbidities, and a history of dysplasia or malignancy 2. This group presents a challenge for physicians, as older adults are often underrepresented in clinical trials, resulting in limited data on treatment outcomes and safety. Drug selection must account for risks such as infections, skin malignancies, lymphoma, and metabolic or cardiovascular complications. Consequently, identifying the optimal drug therapy for managing IBD-related symptoms in this population remains a challenge.

Possibly with fear of abovementioned issues, a recent meta-analysis and systematic review revealed that patients with elderly onset IBD utilize fewer conventional immunomodulator (IMM) and biologic therapies but exhibit comparable or higher rates of IBD-related surgeries than younger patients 3.

No significant differences in efficacy were observed with the use of thiopurines in elderly patients 4, 5. However, there is ongoing debate about the safety of thiopurines in this population. Prescribing thiopurines to elderly patients requires careful evaluation and monitoring due to the potential for adverse events, drug interactions, and an increased risk of lymphoma, non-melanoma skin cancer, and infections 4, 6-10. A recent meta-analysis and systematic review indicated that patients with late-onset IBD tend to use fewer conventional immunomodulators and biologic therapies compared to younger patients, while also exhibiting comparable or even higher rates of IBD-related surgeries 3. It remains unclear whether the differences in the use of IMM therapies and surgery between young-onset and elderly onset IBD indicate a more aggressive disease course in elderly patients or reflect physicians' hesitancy to prescribe IMM and biologics because of concerns about adverse events, comorbidities, polypharmacy, potential drug interactions, or nonadherence in older adults 11.

This study aimed to share the results of IMM treatment in geriatric and non-geriatric patients with IBD and to determine the differences in treatment effectiveness, reasons for treatment discontinuation, and safety profiles between these two cohorts.

## Materials and Methods

### Study design

A retrospective database was established by reviewing the records of IBD patients aged 18 years and older who received IMM therapy at our clinic over a seven-year period. In addition to demographic and clinical data, the Mayo Disease Activity Index and Rachmilewitz Endoscopic Activity Index were used to assess disease activity in patients with UC, whereas the Harvey-Bradshaw Index (HBI) was used for patients with CD. Patients who started IMM therapy treatment at the age of 65 years or older were classified into the geriatric group, and patients receiving IMM younger than 65 years old classified as the non-geriatric group. Covariate adaptive randomization in term of concommitant therapy has been used to collect participants in non-geriatric group. Statistical analyses were performed on the collected data.

Non-response to IMM therapy was defined as a lack of significant symptom reduction, absence of improvement in inflammatory markers, or persistent disease activity on endoscopic evaluation after an adequate treatment period, typically 12-16 weeks for thiopurines. Remission with IMM therapy was defined as the achievement of both clinical and endoscopic remission during treatment.

The current study was conducted at a single tertiary medical center, and the local ethics committee accepted the study (Approval No.: 1-25-900). The study protocol conformed to the ethical guidelines of the 1975 Declaration of Helsinki (sixth revision, 2008), as reflected in the a priori approval of the institution's human research committee.

### Statistical analysis

All analyses were performed using IBM SPSS Statistics for Windows (version 25.0; IBM Corp., Armonk, NY, USA). The Shapiro-Wilk test was used to determine whether the continuous variables were normally distributed. Descriptive statistics are presented using the median (25th percentile-75th percentile) for non-normally distributed continuous variables and frequency (percentage) for categorical variables. Between-group analysis of continuous variables was performed using the Mann-Whitney U test because of the non-normality of distribution. Between-group analysis of categorical variables was performed using the chi-squared test, Fisher's exact test, or Fisher-Freeman-Halton test. Repeated measurements of the scores were analyzed using Friedman's analysis of variance by rank. Pairwise comparisons were adjusted using Bonferroni correction. Two-tailed p-values less than 0.05 were considered statistically significant.

## Results

We included 120 patients with IBD (52 patients with UC and 68 patients with CD) in the study. The median age was 66.5 (23-87, interquartile range 39-71) years, and 64 (53.3%) patients were geriatric (≥65 years). There were no significant differences between geriatric and non-geriatric groups in terms of sex and duration of disease (Table 1).

IMM therapy was discontinued due to side effects in 37 (30.8%) patients. The most common side effects were cytopenia (9, 7.5%), elevated liver function tests (5, 4.2%), and pancreatitis (4, 3.3%). A total of 114 (95.0%) patients were also receiving another treatment. The most common concomitant treatment was mesalamine (100, 83.3%).

Twenty nine (24.2%) patients had no response to IMM therapy (data were not available for 8 patients with CD). IMM therapy was discontinued due to remission in 13 (10.8%) patients. 3 (2.5%) patients developed malignancy during follow-up, and 1 (0.8%) case was mortal. We found no significant differences between geriatric and non-geriatric groups in type of inflammatory bowel disease, duration of IMM therapy, concomitant treatment, nonresponse to IMM therapy, reason for discontinuing the therapy, discontinuation due to remission, discontinuation due to side effects, malignancy, type of malignancy, and mortality (Table 1).

When we evaluated 52 patients with UC, there were no significant differences between geriatric and non-geriatric groups in terms of sex and duration of disease. According to UC types, 4 (7.7%) patients had proctitis, 22 (42.3%) patients had left-sided colitis, 22 (42.3%) patients had extensive colitis, and 4 (7.7%) patients had pancolitis. According to clinical status, 15 (28.8%) patients had a single attack, 10 (19.2%) patients had chronic active colitis, 14 (26.9%) patients had chronic intermittent colitis with rare attacks, and 13 (25.0%) patients had chronic intermittent colitis with frequent attacks. Single attack percentage was significantly higher in the non-geriatric group, while chronic active colitis percentage was higher in the geriatric group (p=0.007) (Table 2).

**Figure 1:**
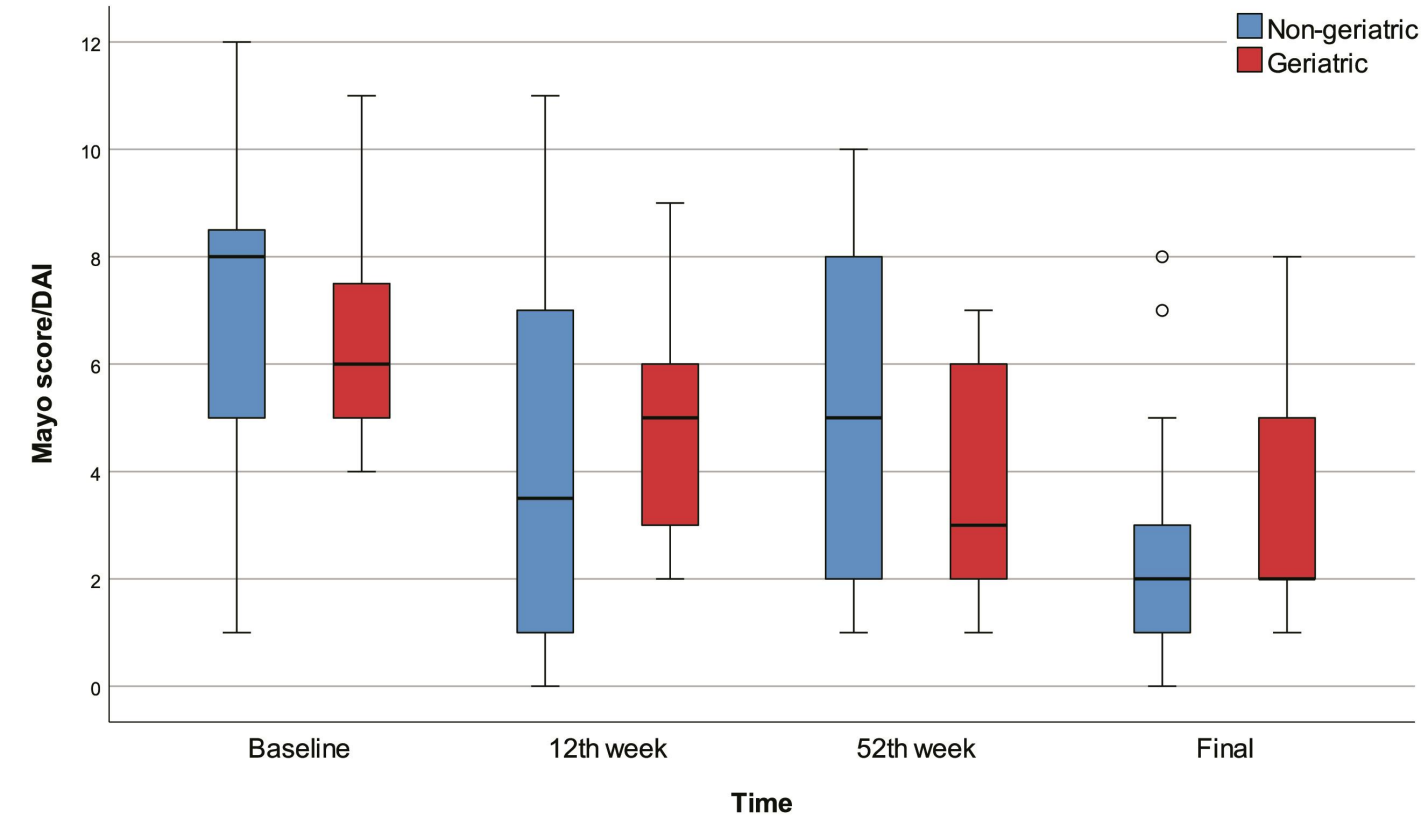
Box-plots of Mayo score / DAI with regard to time and groups.

The 12th week, 52nd week, and final Mayo scores were significantly lower than baseline in all patients (p<0.001). The final Mayo score was significantly lower than baseline in the non-geriatric patients (p=0.001). The 52nd week and final Mayo scores were significantly lower than baseline in the geriatric patients (p<0.001). On the other hand, we found no significant differences between geriatric and non-geriatric groups in terms of baseline, 12th week, 52nd week, final Mayo scores, and change in Mayo score (Figure 1).

**Figure 2:**
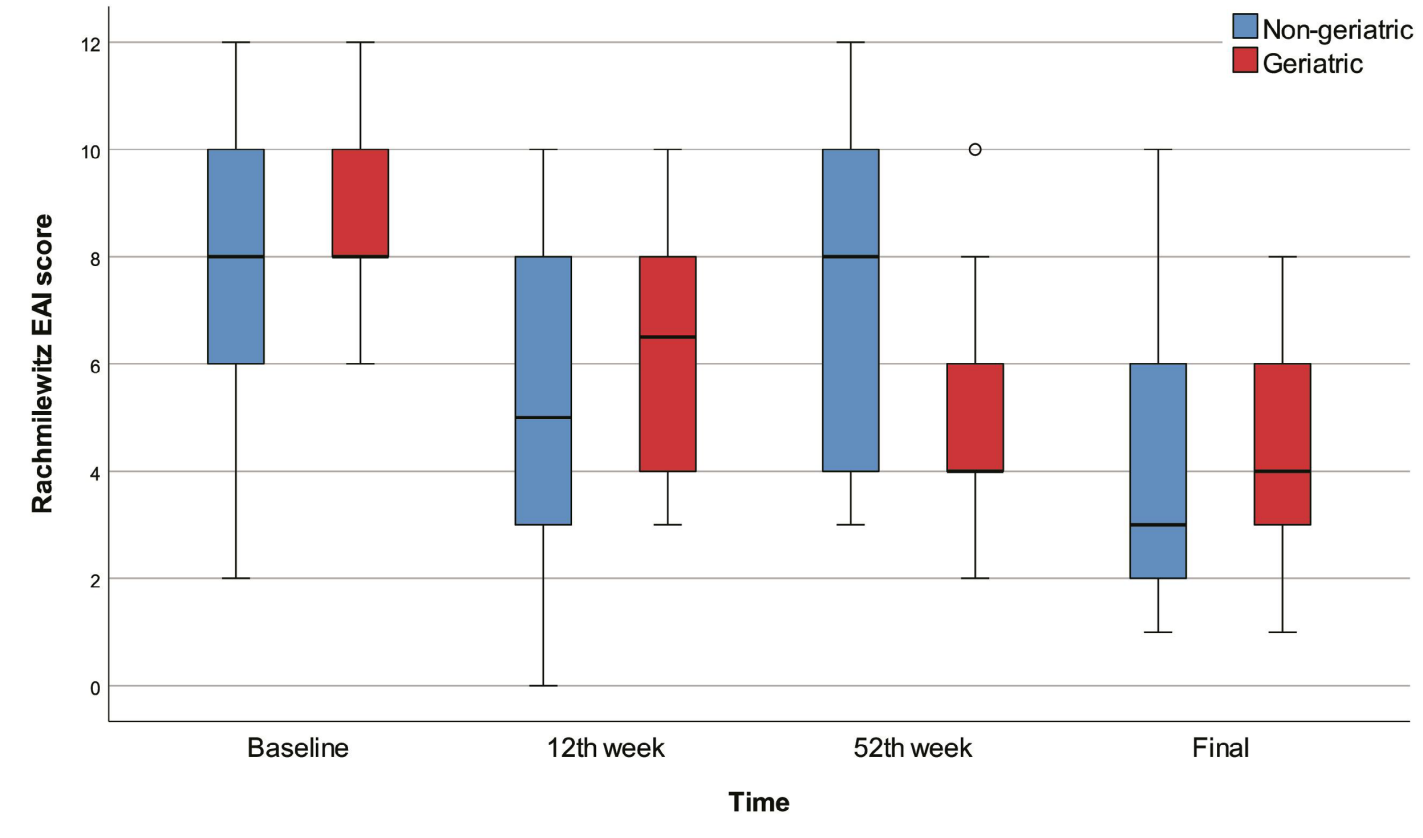
Box-plots of Rachmilewitz score with regard to time and groups.

The 12th week, 52nd week, and final Rachmilewitz scores were significantly lower than baseline in all patients (p<0.001) and in the geriatric patients (p<0.001). There were no significant differences between baseline, 12th week, 52nd week, and final Rachmilewitz scores in the non-geriatric group. On the other hand, we found no significant differences between geriatric and non-geriatric groups in terms of baseline, 12th week, 52nd week, final Rachmilewitz scores and change in Rachmilewitz score. According to the baseline Rachmilewitz score, 42 (94.0%) patients had active disease, while 37 (78.7%) patients had active disease in the 12th week, 34 (82.0%) patients had active disease in the 52nd week, and 26 (61.9%) patients had active disease in the final evaluation. We found no significant differences between geriatric and non-geriatric groups in terms of baseline, 12th week, 52nd week, and final active disease percentages (Table 2, Figure 2).

When we evaluated 68 patients with CD, there were no significant differences between geriatric and non-geriatric groups in terms of sex and duration of disease. 33 (48.5%) patients had inflammatory CD, 26 (38.2%) patients had structuring CD, and 9 (13.2%) patients had penetrating CD. CD was ileal in 23 (33.8%) patients, ileocolonic in 39 (57.4%) patients, and colonic in 6 (8.8%) patients. 11 (16.2%) patients had perianal disease, and 13 (19.1%) patients had resection during IMM therapy (Table 3).

**Figure 3:**
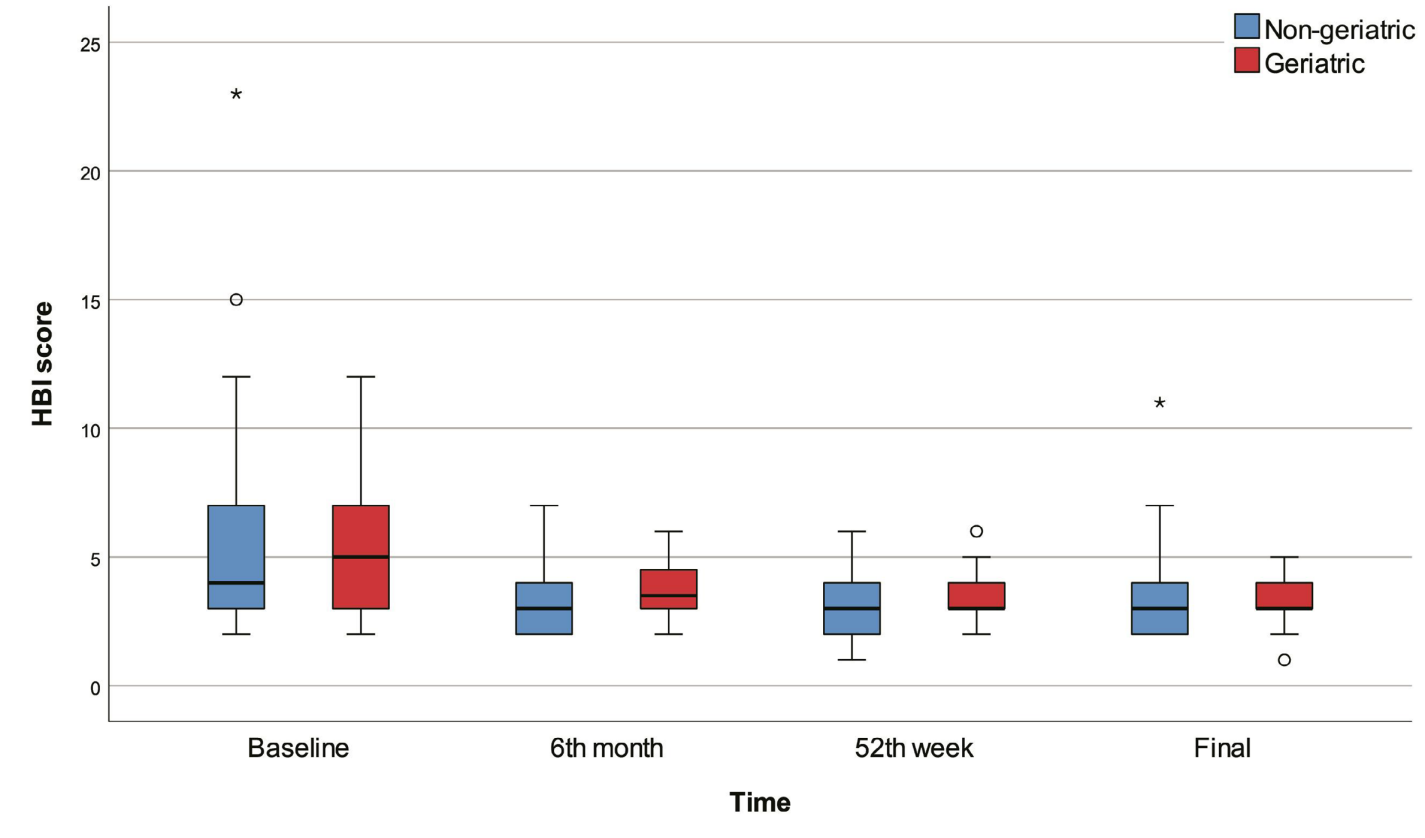
Box-plots of HBI score with regard to time and group.

The 6th month, 52nd week, and final HBI scores were significantly lower than baseline in all patients (p<0.001) and in the non-geriatric patients (p<0.001). The 52nd week and final HBI scores were significantly lower than baseline in the geriatric patients (p<0.001). The 6th month HBI score was significantly higher in the geriatric group than in the non-geriatric group (p=0.015). There were no significant differences between geriatric and non-geriatric groups in terms of baseline, 52nd week, final HBI scores, and change in HBI score (Figure 3).

According to the baseline HBI scores, 7 (10.3%) patients were in remission, while 17 (25.0%) patients were in remission in the 6th month, 15 (22.0%) patients were in remission in the 52nd week, and 19 (28.0%) patients were in remission in the final evaluation. The 6th month (p=0.015) and 52nd week (p=0.023) remission percentages were significantly higher in the non-geriatric group, while mild activity percentages were significantly higher in the geriatric group. There were no significant differences between geriatric and non-geriatric groups in terms of baseline and final remission/active disease percentages.

In addition, the nonresponsiveness to IMM therapy percentage was significantly higher in the non-geriatric group than in the geriatric group (p<0.001). We found no significant differences between geriatric and non-geriatric groups in phenotype of CD, location of CD, perianal disease, duration of IMM therapy, concomitant treatment, reason to discontinue the therapy, discontinuation due to remission, resection during IMM therapy, discontinuation due to side effects, malignancy, type of malignancy, and mortality (Table 3).

## Discussion

IMM therapy was discontinued in 30.8% of patients due to adverse events, 10.8% due to remission, and 24.2% due to non-responsiveness. No significant differences were observed between the geriatric and non-geriatric groups in terms of IBD type, treatment duration, concomitant therapies, treatment response, reasons for discontinuation, adverse events, malignancy, or mortality. Among the 52 UC patients, non-geriatric individuals more frequently presented with single-attack disease, whereas chronic active colitis was more common in the geriatric group (p=0.007). All patients showed significant improvements in Mayo and Rachmilewitz scores over time (p < 0.001), with no significant differences between the groups at baseline or follow-up. In the evaluation of 68 CD patients, the non-geriatric group had a significantly higher rate of non-responsiveness to IMM therapy (p<0.001). However, there were no significant differences between the two groups regarding CD phenotype, location, perianal disease, treatment duration, reasons for discontinuation, side effects, malignancy, or mortality. Additionally, no significant differences were observed in baseline or follow-up HBI scores, except for higher 6th month HBI scores and lower remission rates in the geriatric group.

In the last decade, recommendations and guidelines for the management of geriatric patient groups have been published due to the physiological and pathological differences in the geriatric patient group 9, 12-15. Although these guidelines provide recommendations for IMM therapy, its use in geriatric patients remains a topic without an absolute recommendation. The fact that IMM treatment alternatives are not innocent makes it difficult to give up treatment immediately, particularly in geriatric patients. The decrease rate in IMM prescriptions in the elderly is consistent with many studies 9,10. However, Kariyawasam et al. showed that age-related factors including comorbid status rather than age itself, were associated with IMM use. Comorbidity was shown to delay the time at which IMMs were introduced in both CD and UC, whereas age at diagnosis was not associated with IMM introduction 16.

For steroid-sparing therapy, physicians commonly use 6-mercaptopurine or azathioprine. Research indicates that the effectiveness of these IMM therapies does not differ significantly between patients aged > 60 years and those aged < 60 13. Similarly, we have found that IMM therapy effectiveness was comparable between geriatric and non-geriatric patients.

Substantial evidence shows that using thiopurines in elderly patients with IBD increases the risk of infections, lymphoproliferative disorders, and skin cancers. However, it should be kept on mind that the incidence of these morbidities are also increased in geriatric population regardless of treatment 17-19. In our study population, the frequency of these complications did not differ between study groups. It is known that the use of anti-TNF in combination with IMM increases this risk even more. In our study, we believe that the very low number of patients using the IMM + anti-TNF combination in both groups caused this result.

The safety outcomes of anti-TNF treatment, which is considered the preferred option over IMM, are also evident. In a cohort study of 734 IBD patients treated with anti-TNF therapy, compared with 666 patients receiving other treatments, age at the initiation of anti-TNF therapy was the sole independent predictor of mortality 20. Desai et al. 21 similarly reported a threefold higher risk of anti-TNF discontinuation in patients starting therapy after age 60 compared to older azathioprine users. In a multicenter observational study, Cottone et al. 22 found that elderly patients receiving biologic treatments (infliximab [n = 2475] or adalimumab [n = 604]) had an increased risk of infection, malignancy, and mortality compared to younger patients (13% vs. 2.6%, 3% vs. 0%, and 10% vs. 1%, respectively) and elderly patients receiving other therapies. A recent study from Leuven echoed these findings, noting that patients over 65 on anti-TNF therapy had a significantly higher risk of severe adverse events, with malignancy and infections more prevalent in this group (relative risk = 4.7; p < 0.001) 23.

The availability of newer agents, such as IL-23 inhibitors, anti-integrins, and JAK inhibitors, offers promising alternative treatment options for anti-TNF or IMM therapy. However, evidence supporting the specific use of IL-23 inhibitors in elderly patients with IBD remains limited, although their safety profile may be suitable for elderly individuals with comorbidities. Owing to the elevated risk of major cardiovascular events, venous thromboembolism, and malignancy, major regulatory agencies recommend avoiding JAK inhibitors in patients over 65 years of age unless no other viable treatment options are available 24.

The main limitations of our study include the relatively small sample size, its retrospective design, and the short duration of IMM therapy. In addition, detailed baseline comorbidity data (such as hypertension, diabetes, cardiovascular or pulmonary disease) were not systematically recorded in our database, which limited our ability to assess their potential impact, particularly in the elderly cohort. Nevertheless, an important strength is the balanced patient groups with comparable disease durations between the geriatric and non-geriatric cohorts. Furthermore, only four patients in the IMM group were also receiving concomitant anti-TNF therapy, while the vast majority (95.8%) were not, thereby minimizing the confounding effect of combination therapy and the increased risk of adverse events commonly associated with concurrent IMM and anti-TNF use 19.

In conclusion, this study highlights that IMM therapy can be a feasible option for elderly IBD patients when selected and monitored carefully, showing comparable efficacy and safety profiles to younger patient groups. No significant differences were found between the geriatric and non-geriatric groups in terms of disease type, treatment duration, response rates, adverse events, or malignancy risks, potentially because of balanced disease durations and appropriate screening and follow-up. Future studies with larger cohorts and longer follow-up periods are needed to further clarify the role and safety profile of IMM therapy in elderly patients with IBD.

## Author contributions

D.A.: Conception and design of the study, analysis and interpretation of data, and revision of the manuscript. K.K.: Study design, data collection, data analysis and interpretation, and article writing. Y.Ö.Ö.: Conception and design of the work. F.B: Study design, data collection. V.G: Study design, data collection. S.A: Study design, data collection. Ö.Ö: Conception and design of the study, analysis and interpretation of data. İ.H.K.: Collecting data, analysis and interpretation of data, and revising the article.

## Ethical approval

Ethical approval was obtained from the ethics committee of Ankara Bilkent City Hospital.

## Informed consent

Written informed consent was obtained from all patients before inclusion.

## Availability of data

The data are available upon request from the authors.

## Figures and Tables

**Table 1:** Demographics, disease and treatment characteristics of the study population

Variables	Total (n=120)	Non-geriatric (n=56)	Geriatric (n=64)	p
Age, years (median, range)	66.5 (23-87)	37 (29-49)	70 (68-74)	<0.05^‡^
Sex, n (%)				
Female	36 (30.0%)	21 (37.5%)	15 (23.4%)	≥0.05^§^
Male	84 (70.0%)	35 (62.5%)	49 (76.6%)	
Duration of disease, months (median, range)	68 (34-108)	71 (29-101)	65 (38-111)	≥0.05^‡^
Inflammatory bowel disease type, n (%)				
Ulcerative colitis	52 (43.3%)	22 (39.3%)	30 (46.9%)	≥0.05^§^
Crohn's disease	68 (56.7%)	34 (60.7%)	34 (53.1%)	
Age at diagnosis, years (median, range)	61 (29-66)	29 (25-41)	66 (64-68)	<0.05^‡^
Age at IMM onset, years (median, range)	65 (32-68)	31 (27-42)	67 (66-69)	<0.05^‡^
Concomitant treatment, n (%)				
None	6 (5.0%)	3 (5.4%)	3 (4.7%)	≥0.05^¶^
Mesalamine	100 (83.3%)	43 (76.8%)	57 (89.1%)	
Sulfasalazine	4 (3.3%)	2 (3.6%)	2 (3.1%)	
Anti-TNF	2 (1.7%)	0 (0.0%)	2 (3.1%)	
Mesalamine + Prednisolone	5 (4.2%)	4 (7.1%)	1 (1.6%)	
Mesalamine + Anti-TNF	3 (2.5%)	2 (3.6%)	1 (1.6%)	
Duration of IMM therapy, months (median, range)	23 (12-55)	28 (13-54)	20 (12-60)	≥0.05^‡^
Unresponsive to IMM therapy, n (%)	29 (24.2%)	18 (32.1%)	11 (17.2%)	≥0.05^§^
Discontinuation due to remission, n (%)	13 (10.8%)	4 (7.1%)	9 (14.1%)	≥0.05^§^
Discontinuation due to side effect, n (%)	37 (30.8%)	20 (35.7%)	17 (26.6%)	≥0.05^§^
Pancreatitis	4 (3.3%)	2 (3.6%)	2 (3.1%)	≥0.05^¶^
Cytopenia	9 (7.5%)	5 (8.9%)	4 (6.3%)	
Elevated liver function tests	5 (4.2%)	2 (3.6%)	3 (4.7%)	
Other	19 (15.8%)	9 (16.1%)	10 (15.6%)	
Malignancy during follow-up, n (%)	3 (2.5%)	0 (0.0%)	3 (4.7%)	
Lymphoproliferative	1 (0.8%)	0 (0.0%)	1 (1.6%)	≥0.05^#^
Solid	2 (1.7%)	0 (0.0%)	2 (3.1%)	
Mortality, n (%)	1 (0.8%)	0 (0.0%)	1 (1.6%)	≥0.05^#^

Values are median (range) for continuous variables and n (%) for categorical variables. Statistical comparisons between geriatric and non-geriatric groups were performed using: ‡Mann-Whitney U test; §Chi-square test; #Fisher's exact test; ¶Fisher-Freeman-Halton test. p<0.05 was considered statistically significant.

**Table 2:** Demographics, disease and treatment characteristics in ulcerative colitis patients

Variables	Total (n=52)	Non-geriatric (n=22)	Geriatric (n=30)	p
Age, years (median, range)	67 (35-74)	45 (33-57)	70 (69-74)	<0.05^‡^
Sex, n (%)				
Female	12 (23.1%)	7 (31.8%)	5 (16.7%)	≥0.05^#^
Male	40 (76.9%)	15 (68.2%)	25 (83.3%)	
Type of UC, n (%)				
Proctitis	4 (7.7%)	2 (9.1%)	2 (6.7%)	0.05^¶^
Left-sided colitis	22 (42.3%)	7 (31.8%)	15 (50.0%)	
Extensive	22 (42.3%)	9 (40.9%)	13 (43.3%)	
Pancolitis	4 (7.7%)	4 (18.2%)	0 (0.0%)	
Clinical status of UC, n (%)				
Single attack	15 (28.8%)	10 (45.5%)	5 (16.7%)	<0.05^¶^
Chronic active colitis	10 (19.2%)	0 (0.0%)	10 (33.3%)	
Chronic intermittent, rare attack	14 (26.9%)	5 (22.7%)	9 (30.0%)	
Chronic intermittent, frequent attack	13 (25.0%)	7 (31.8%)	6 (20.0%)	
Duration of disease, months (median, range)	75 (37-125)	85 (49-150)	63 (36-118)	≥0.05^‡^
Age at diagnosis, years (median, range)	62 (38-66)	34 (26-47)	66 (64-68)	<0.05^‡^
Age at IMM onset, years (median, range)	65 (46-68)	40 (32-53)	67 (66-69)	<0.05^‡^
Concomitant treatment, n (%)				
None	1 (1.9%)	1 (4.5%)	0 (0.0%)	≥0.05^¶^
Mesalamine	44 (84.6%)	15 (68.2%)	29 (96.7%)	
Mesalamine + Prednisolone	5 (9.6%)	4 (18.2%)	1 (3.3%)	
Mesalamine + Anti-TNF	2 (3.8%)	2 (9.1%)	0 (0.0%)	
Duration of IMM therapy, months (median, range)	22 (12-48)	24 (10-46)	16 (12-50)	≥0.05^‡^
Unresponsive to IMM therapy, n (%)	15 (28.8%)	6 (27.3%)	9 (30.0%)	≥0.05^§^
Discontinuation due to remission, n (%)	6 (11.5%)	1 (4.5%)	5 (16.7%)	≥0.05^#^
Discontinuation due to side effect, n (%)	16 (30.8%)	8 (36.4%)	8 (26.7%)	≥0.05^§^
Pancreatitis	1 (1.9%)	0 (0.0%)	1 (3.3%)	≥0.05^¶^
Cytopenia	4 (7.7%)	2 (9.1%)	2 (6.7%)	
Elevated liver function tests	3 (5.8%)	2 (9.1%)	1 (3.3%)	
Other	8 (15.4%)	3 (13.6%)	5 (16.7%)	
Malignancy during follow-up, n (%)	1 (1.9%)	0 (0.0%)	1 (3.3%)	≥0.05^#^
Mortality, n (%)	1 (1.9%)	0 (0.0%)	1 (3.3%)	≥0.05^#^

Values are median (range) for continuous variables and n (%) for categorical variables. Statistical comparisons between geriatric and non-geriatric groups were performed using: ‡Mann-Whitney U test; §Chi-square test; #Fisher's exact test; ¶Fisher-Freeman-Halton test. p<0.05 was considered statistically significant.

**Table 3:** Demographics, disease and treatment characteristics in Crohn's disease patients

Variables	Total (n=68)	Non-geriatric (n=34)	Geriatric (n=34)	p
Age, years (median, range)	66 (31-73)	32 (28-43)	71 (68-73)	<0.05‡
Sex, n (%)				
Female	24 (35.3%)	14 (41.2%)	10 (29.4%)	≥0.05§
Male	44 (64.7%)	20 (58.8%)	24 (70.6%)	
Duration of disease, months (median, range)	65 (34-92)	61 (27-88)	75 (46-98)	≥0.05‡
Age at diagnosis, years (median, range)	57 (27-66)	28 (25-35)	65 (63-66)	<0.05‡
Location of CD, n (%)				≥0.05¶
Ileal	23 (33.8%)	13 (38.2%)	10 (29.4%)	≥0.05¶
Ileocolonic	39 (57.4%)	18 (52.9%)	21 (61.8%)	
Colonic	6 (8.8%)	2 (5.9%)	4 (11.8%)	
Phenotype of CD, n (%)				≥0.05¶
Inflammatory	33 (48.5%)	15 (44.1%)	18 (52.9%)	≥0.05¶
Structuring	26 (38.2%)	13 (38.2%)	13 (38.2%)	
Penetrating	9 (13.2%)	6 (17.6%)	3 (8.8%)	
Perianal disease, n (%)	11 (16.2%)	6 (17.6%)	5 (14.7%)	≥0.05#
Age at IMM onset, years (median, range)	59 (28-67)	29 (26-34)	66 (66-69)	<0.05‡
Concomitant treatment, n (%)				
None	4 (5.9%)	2 (5.9%)	2 (5.9%)	≥0.05¶
Mesalamine	56 (82.4%)	28 (82.4%)	28 (82.4%)	
Sulfasalazine	3 (4.4%)	2 (5.9%)	1 (2.9%)	
Anti-TNF	2 (2.9%)	0 (0.0%)	2 (5.9%)	
Mesalamine + Anti-TNF	3 (4.4%)	2 (5.9%)	1 (2.9%)	
Duration of IMM therapy, months (median, range)	26 (13-62)	34 (18-69)	21 (13-61)	≥0.05‡
Unresponsive to IMM therapy, n (%)	14 (20.6%)	12 (35.3%)	2 (5.9%)	<0.05#
Discontinuation due to remission, n (%)	7 (10.3%)	2 (5.9%)	5 (14.7%)	≥0.05#
Resection during IMM therapy, n (%)	13 (19.1%)	5 (14.7%)	8 (23.5%)	≥0.05§
Discontinuation due to side effect, n (%)	21 (30.9%)	11 (32.4%)	10 (29.4%)	≥0.05§
Pancreatitis	3 (4.4%)	2 (5.9%)	1 (2.9%)	≥0.05¶
Cytopenia	5 (7.4%)	3 (8.8%)	2 (5.9%)	
Elevated liver function tests	2 (2.9%)	1 (2.9%)	1 (2.9%)	
Other	11 (16.2%)	5 (14.7%)	6 (17.6%)	
Malignancy during follow-up, n (%)	2 (2.9%)	0 (0.0%)	2 (5.9%)	≥0.05#
Mortality, n (%)	0 (0.0%)	0 (0.0%)	0 (0.0%)	—

Values are median (range) for continuous variables and n (%) for categorical variables. Statistical comparisons between geriatric and non-geriatric groups were performed using: ‡Mann-Whitney U test; §Chi-square test; #Fisher's exact test; ¶Fisher-Freeman-Halton test. p<0.05 was considered statistically significant.
